# Education Research: The Inappropriate Consult: Discordant Expectations of Specialty Expertise and Areas for Improvement in Interdisciplinary Resident Education

**DOI:** 10.1212/NE9.0000000000200044

**Published:** 2023-01-10

**Authors:** Charles Sanky, Caroline Gentile, Jennifer Ren, Eric Bortnick, Stephen Krieger

**Affiliations:** From the Department of Emergency Medicine (C.S.), and Department of Medical Education (C.S., J.R., E.B.), Icahn School of Medicine at Mount Sinai, New York, NY; Department of Neurology (C.G.), University of Pennsylvania, Philadelphia; and Department of Neurology (S.K.), Icahn School of Medicine at Mount Sinai, New York, NY.

## Abstract

**Background and Objectives:**

As resident physicians specialize, they lose familiarity with knowledge central to other fields. This can yield what we term the dual fallacies: (1) the sense that their own expertise is common knowledge, and (2) unfamiliar clinical situations seem beyond their scope. In graduate medical education, these dual fallacies may engender the perception of inappropriate consults among specialties. This project evaluated biases in residents' perceptions of expected knowledge and inappropriate consults to improve interdisciplinary education among neurology residents (neurologists) and internal medicine residents (internists). Secondarily, we evaluated whether these biases were mitigated after implementing an educational intervention.

**Methods:**

Resident neurologists and internists at a large, urban, academic medical center answered board-style questions reflecting neurology and medicine consultation scenarios. They then rated the extent to which each scenario reflected common knowledge to both specialties and whether a consult was warranted. After revising the internal medicine residency curriculum to include a neurology rotation, another cohort of residents was surveyed and participated in semistructured interviews. Paired sample *t* tests and qualitative data analysis were performed.

**Results:**

Neurologists and internists participated in phase 1 (n = 23) and phase 2 (n = 42) of the study. Residents from both fields answered more questions correctly from their own specialty than the other in phase 1 (*p* < 0.05) and phase 2 (*p* < 0.001). Neurologists and internists in both cohorts thought that each other should know more neurology answers than they actually did (*p* < 0.05). Neurologists were less likely to agree than internists that medicine questions deserved a consult (*p* = 0.014). Interviews revealed themes regarding perceived consult appropriateness, affected by educational, communication, clinical, and administrative factors. In addition, residents agreed that appropriate consults must pose a specific question and occur only after an initial investigation was performed, but that this rarely happens.

**Discussion:**

Our findings support that discordant expectations of expertise contribute to a perception of inappropriate consults among neurologists. Nonclinical factors, from cognitive biases to contextual considerations, inform clinical consultation and interdisciplinary patient care. Implementing rotations on other services alone is insufficient to eradicate discordant expectations; however, we propose additional interventions that may prove valuable in medical education.

Consultation, in which a physician helps another by offering their expertise, is integral to communication and patient care among physicians of different specialties. The ten commandments for effective consultation defined characteristics of ideal consults and highlighted the need for clear communication.^1^ In academic medical systems, resident physicians are typically responsible for calling and responding to consults. Effective consultation is so important that the Accreditation Council for Graduate Medical Education's core competencies explicitly stipulate that residents should be able to seek additional guidance and/or consultation as appropriate, effectively communicate with other physicians, other health professionals, and as members of interdisciplinary teams, and act in a consultative role to other physicians and health professionals.^2^ Residents are also responsible for identifying strengths, deficiencies, and limits in one's knowledge and expertise, which is integral to appropriate consultation. Furthermore, residents across disciplines believe that consultation greatly affect patient care.^3^

Despite these guidelines and beliefs, there is a pervasive sense that some consults are inappropriate, time wasting, and someone's attempt to relieve their own responsibilities. A recent comedic TikTok video series by an ophthalmologist aliased Dr. Glaucomflecken distills each discipline's core beliefs and identifies how subcultures misunderstand each other.^4^ Particularly, his character, The Neurologist, comments on the inability of other specialties to perform a neurologic examination of patients, localization of pathology, and conflicting approaches to common chief consults such as altered mental status. Accumulating 18.7 million likes in a year, these videos have amassed popularity among the medical community, as this issue resonates with those in medical education and inpatient practice. Mismatched expectations of clinical expertise can lead to poor communication and negative effects on patient safety.^5^ Inappropriate consults also minimize learning opportunities, which is deleterious in residency programs designed to teach neurology resident physicians effective interdisciplinary collaboration.

Although prior studies surveyed residents about perceptions of consults and subsequently developed training and informational cards,^3^ little attention has been paid to identifying how and why residents deemed a consult appropriate. A prior study by our team evaluated real-time perceptions of consult appropriateness between practitioners who call neurology consults and neurology residents.^6^ Although our prior investigation identified differing expectations by providers on both sides of the consults, the purpose of this study is to explore the cognitive biases and structural factors that drive these discordant perceptions.

Possible explanations for seemingly inappropriate consults include that as physicians subspecialize, they begin to consider their own knowledge as common sense while losing familiarity with knowledge central to other fields. This leads to what we have termed the dual fallacies: although physicians perceive that their expertise is common knowledge shared among other specialties, they erroneously characterize unfamiliar clinical scenarios as obscure or beyond their scope. Although this phenomenon has not been investigated in the medical field, social psychology research has explored both components. Reflecting the first of the dual fallacies, knowledgeable individuals overestimate the identifiability of their own knowledge, while in fact, what is seemingly obvious to 1 person is rarely obvious to another.^7^ These dual fallacies yield potential areas of overlap and neglect among specialties with respect to what clinical presentations should belong to different fields, thus engendering the pervasive perception of inappropriate consults. To explore the common sentiment of inappropriate consultation by neurology residents, this project sought to assess biases in perceived knowledge base, measure effects on residents' perception of inappropriate consults, and examine the effect of educational efforts to bridge perceived gaps among specialties.

## Methods

### Setting

This study was conducted across 4 years at a large urban academic medical center in 2 phases, between which a rotation for internal medicine residents on the neurology consult service was implemented. Our primary objective was to evaluate whether discordant perceptions of knowledge drive biases in interdisciplinary consultation. Secondarily, we sought to assess whether the educational intervention reduced these discordant perceptions of knowledge by comparing phase 1 to 2. We compared neurology residents (neurologists) and internal medicine residents (internists) because they are related but distinct specialties, whereas internal medicine residents spend 3 years training in internal medicine, and neurology residents spend 1 year in internal medicine before commencing their specialized neurology training. By comparing a foundational, generalized discipline to a medical subspecialty (neurology), we examine whether the dual fallacies exist with subspecialization; we anticipate further discordance between specialties without this overlap. Phase 1's objective was to develop our survey questions and methodology, and phase 2's objective was to replicate the augmented survey and conduct semistructured interviews with a new cohort. We used a sequential transformative mixed-methods approach^8^; our dual fallacy principle provides a theoretical framework, our board-style questions and perception assessments provide quantitative data, and our semistructured interviews provide qualitative data and contextualize the findings. Quantitative methods assessed participants' knowledge and perceptions, whereas qualitative methods probed the motivations behind, attitudes toward, and hopes for consultation.

### Standard Protocol Approvals, Registrations, and Patient Consents

Both phases were submitted to our institutional review board and deemed exempt due to interactions involving interview procedures with data that are deidentified (45 CFR 46.104(d)(2)). All data from resident participants were deidentified.

### Phase 1

The objectives for phase 1 were to test our survey and methodology for measuring residents' knowledge bases, identify residents' perceptions of knowledge bases between the 2 specialties, and assess whether residents felt that a scenario warranted a consult. The survey collected demographic information, prompted participants to self-rate their knowledge in both internal medicine and neurology, and presented 5 neurology and 5 medicine board-style questions reflecting common consultation scenarios. These vignettes (sample questions in eTable 1, links.lww.com/NE9/A15) were drafted by a committee of senior residents in both specialties to reflect common inpatient scenarios, and they were refined by the senior author, the Program Director of the neurology residency program. For each scenario, residents answered a question to assess their knowledge and rated whether each scenario should be common knowledge, was actually common knowledge, and warranted a consult. Outcome measures included Likert scale responses to each question. Table 1 reflects participants' demographics.

**Table 1:** Study Participants

	Phase 1 (n = 23)	Phase 2 survey (n = 42)
Internal medicine residents (internists)	n = 10 (43.5%)	n = 24 (57.1%)
Neurology residents (neurologists)	n = 13 (56.5%)	n = 18 (42.9%)
Male	n = 14 (60.9%)	n = 15 (35.7%)
Female	n = 9 (39.1%)	n = 27 (64.3%)
PGY-2	n = 0 (0%)	IMR: n = 10 (41.7%) NR: n = 7 (38.9%)
PGY-3	Internists: n = 8 (80%) Neurologists: n = 7 (53.8%)	Internists: n = 14 (58.3%) Neurologists: n = 5 (27.8%)
PGY-4	Internists: n = 2 (20%) Neurologists: n = 6 (46.2%)	Internists: n = 0 (0%) Neurologists: n = 6 (33.3%)

Abbreviation: PGY = postgraduate year.

After phase 1, our institution created a rotation for internal medicine residents on the neurology consult service for 2 weeks during intern year (postgraduate year [PGY]-1). Through this educational intervention, internists were exposed to common neurologic consultation scenarios and had the opportunity to understand neurology's diagnostic and management approaches. We also gathered feedback from neurologists and internists on the questions' appropriateness and difficulty to validate these questions for phase 2. We consequently added questions assessing residents' confidence in calling consults and whether their residency program included education in other specialties.

### Phase 2

The objectives for phase 2 were twofold: we again assessed whether interdisciplinary consultations are affected by discordant expectations of specialty expertise with a new cohort, and second, we sought to evaluate whether the educational intervention (neurology rotation for PGY-1 internists) affected outcomes. This second phase surveyed a different cohort of neurologists and internists 3 years after the neurology rotation's implementation, so all phase 2 internists had rotated through the neurology service. Outcome measures included Likert scale responses to each question. Finally, we compensated busy residents with gift cards to increase participation rates compared with phase 1. Again, Table 1 reflects participants' demographics. Because of structural changes in our neurology residency program, PGY-2s were included in phase 2 because they were more involved in consultation than in previous years.

### Semistructured Interviews

Phase 2 survey participants were given the option to participate in a semistructured interview to further explore their attitudes toward consultations. We collected responses until we achieved thematic saturation, which was rooted in a grounded theory model of qualitative data.^9^ We found that we were able to achieve thematic saturation after 7 internist interviews and 7 neurologist interviews, for a total of 14 interviews. Participants were predominantly PGY-3 residents who had served on their specialty's consult service. For 30 minutes, participants were individually asked 9 standard questions (eTable 2, links.lww.com/NE9/A15) and then permitted to direct the conversation with examples from their clinical practice. These questions were developed from literature review on cognitive biases in clinical practice, discussion with residents and faculty in both specialties, and after analysis and interpretation of data from the survey components of this study.

### Data Analysis

For surveys, SPSS was used to characterize data, namely demonstrating through analysis of variance that data were parametric. Thus, paired sample *t* tests were used in both phases to understand differences between residents of both specialties.

Semistructured interviews were audiotaped and transcribed verbatim, immediately after the sessions were completed to ensure data quality and integrity. We conducted an initial round of coding using thematic analysis stemming from grounded theory qualitative analysis. Codes were developed in an iterative approach as data were analyzed. Triangulation of qualitative data with quantitative data from the 2 phases of the research study was used. We then refined codes to organize themes and subthemes among the data. Two blinded researchers independently conducted qualitative, thematic data analysis using NVivo software to elucidate findings of structured interviews.

### Data Availability

All anonymized data not published within the article are available and may be shared by request from any qualified investigator.

## Results

### Phase 1

Thirteen neurologists and 10 internists completed the phase 1 survey (n = 23). Paired sample *t* tests revealed that internists correctly answered significantly more medicine than neurology questions (3.80 vs 1.90; 95% CI 1.189–2.611; *p* < 0.001) (Figure). In addition, neurologists answered significantly more neurology questions correctly than internists (2.85 vs 1.90; 95% CI 0.236–1.656; *p* = 0.011).

**Figure:**
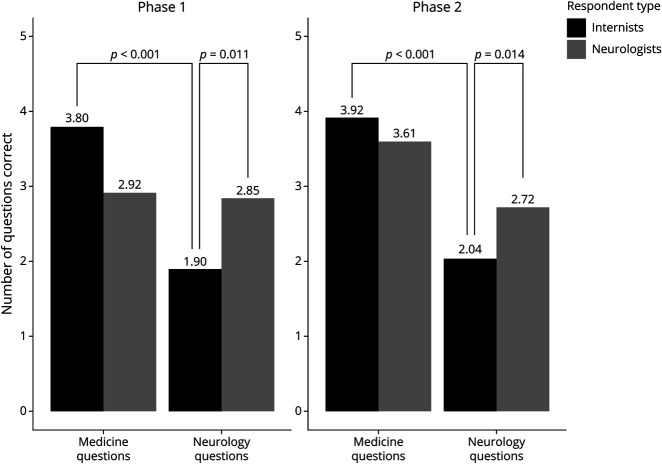
Average Number of Questions Correct, by Specialty and Question Type Statistically significant comparisons demonstrated with *p* < 0.05.

We next probed whether each scenario warranted a consult (Table 2). Although internists and neurologists agreed that neurology consultation was appropriate for neurology scenarios (3.54 vs 3.68; 95% CI −0.539 to 0.266; *p* = 0.501), neurologists were less likely to agree that medicine questions deserved a consult (2.69 vs 3.22; 95% CI 0.108–0.947; *p* = 0.014).

**Table 2:** Perceptions of Consult Appropriateness

	Internists Mean (SD)	Neurologists Mean (SD)	Absolute difference (CI)	*p* Value
Phase 1				
Is this an appropriate neurology consult?	3.54 (1.16)	3.68 (0.95)	0.14 (−0.266 to 0.539)	0.501
Is this an appropriate internal medicine consult?	3.22 (1.13)	2.69 (1.12)	0.53 (0.108 to 0.947)	0.014
Phase 2				
Is this an appropriate neurology consult?	3.38 (0.55)	3.67 (0.41)	0.29 (−0.087 to 2.921)	0.064
Is this an appropriate internal medicine consult?	2.58 (0.72)	2.19 (0.60)	0.39 (−0.122 to 3.983)	0.065

Finally, we measured residents' perceptions of the 2 specialties' knowledge bases (Table 3). Internists thought that neurologists should know more neurology answers than neurologists actually did (4.48 vs 4.28; 95% CI 0.072–0.328; *p* = 0.003). Conversely, neurologists thought that internists should know more neurology answers than internists actually did (3.17 vs 3.02, 95% CI 0.001–0.312; *p* = 0.049). Contrary to expectations, internists were more likely than neurologists to state that internists should know more neurology answers (3.82 vs 3.17; 95% CI 0.357–0.976; *p* < 0.001). Members of both groups thought that they should know more neurology answers than they actually did (*p* ≤ 0.001 for both).

**Table 3:** Phase 1 Perceptions of Specialty Knowledge

	Internists should know the answer Mean (SD)	Internists would know the answer Mean (SD)	Absolute difference (CI)	*p* Value	Neurologists should know the answer Mean (SD)	Neurologists would know the answer Mean (SD)	Absolute difference (CI)	*p* Value
Neurology questions								
Internists	3.82 (0.73)	3.45 (0.94)	0.37 (0.167 to 0.568)	0.001^a^	4.48 (0.58)	4.28 (0.54)	0.20 (0.072 to 0.328)	0.003^a^
Neurologists	3.17 (0.96)	3.02 (0.86)	0.15 (0.001 to 0.312)	0.049^a^	4.38 (0.63)	4.20 (0.62)	0.18 (0.088 to 0.282)	<0.001^a^
Medicine questions								
Internists	4.48 (0.61)	4.34 (0.56)	0.14 (−0.001 to 0.281)	0.051	3.60 (0.90)	3.48 (0.97)	0.12 (−0.067 to 0.307)	0.204
Neurologists	4.42 (0.56)	4.25 (0.62)	0.17 (0.058 to 0.286)	0.004^a^	3.83 (0.84)	3.69 (0.84)	0.014 (0.001 to 0.276)	0.049^a^

a Statistically significant at *p* < 0.05.

### Phase 2

We again measured residents' knowledge bases among 18 neurologists and 24 internists (n = 42) and found concordant evidence to phase 1. Specifically, neurologists and internists again answered significantly more questions correctly from their own specialty than from the other field (*p* < 0.001), and neurologists answered significantly more neurology questions correctly than internists (2.72 vs 2.04; 95% CI 0.152–1.199; *p* = 0.014) (Figure).

There was, again, no statistically significant difference between neurologists and internists as to whether neurology consultation was appropriate for neurology scenarios (3.67 vs 3.38; 95% CI −0.087 to 2.921; *p* = 0.064) (Table 2). Unlike the results of phase 1, there was no statistically significant difference between groups regarding medicine consult appropriateness for medicine questions (2.19 vs 2.58; 95% CI −0.122 to 3.983; *p* = 0.065).

We retested residents' perceptions of each other's knowledge bases (Table 4). Consistent with phase 1's outcomes, both neurologists and internists expected that the other should know more about neurology than they would actually know; internists thought that neurologists should know more neurology information than neurologists actually would know (4.55 vs 4.37; 95% CI 0.057–0.310; *p* = 0.007), and neurologists thought that internists should know more neurology than internists actually would know (3.04 vs 2.77; 95% CI 0.009–0.547; *p* = 0.044).

**Table 4:** Phase 2 Perceptions of Specialty Knowledge

	Internists should know the answer Mean (SD)	Internists would know the answer Mean (SD)	Absolute difference (CI)	*p* Value	Neurologists should know the answer Mean (SD)	Neurologists would know the answer Mean (SD)	Absolute difference (CI)	*p* Value
Neurology questions								
Internists	3.63 (0.41)	3.49 (0.35)	0.14 (−0.013 to 0.280)	0.073^a^	4.55 (0.38)	4.37 (0.40)	0.18 (0.057 to 0.310)	0.007^a^
Neurologists	3.04 (0.52)	2.77 (0.52)	0.31 (0.009 to 0.547)	0.044^a^	4.42 (0.40)	4.30 (0.41)	0.12 (0.031 to 0.213)	0.012^a^
Medicine questions								
Internists	4.64 (0.38)	4.53 (0.36)	0.11 (0.009 to 0.208)	0.034^a^	3.71 (0.50)	3.54 (0.41)	0.17 (−0.026 to 0.359)	0.086
Neurologists	4.60 (0.41)	4.58 (0.42)	0.02 (−0.025 to 0.069)	0.331	4.02 (0.57)	3.96 (0.61)	0.06 (−0.065 to 0.199)	0.302

a Statistically significant at *p* < 0.05.

Although both groups of residents highly rated their own abilities to discern an appropriate consult, internists scored significantly higher than neurologists (4.38 vs 3.89; 95% CI 0.032–0.940; *p* = 0.037), indicating that internists believed that they were more capable of discerning consult appropriateness. In concordance with phase 1's results, internists were more likely than neurologists to feel that internists should know the answer to neurology questions. Internists felt that their programs provided more education in other specialty areas than neurologists (3.88 vs 2.61; 95% CI 0.675–1.853; *p* = 0.021).

### Semistructured Interviews

Semistructured interviews interrogated the anatomy of a good consult and what could improve consult behavior. Five themes emerged for whether a consult is perceived as appropriate: (1) consults should pose a specific question; (2) the requester should complete an initial investigation; (3) consults also be an educational opportunity; (4) attendings call consults to reduce liability; and (5) consult quality is affected by workload (Table 5). Themes of educational, communication, clinical, and administrative factors affecting consult appropriateness were identified (κ coefficient of interrater reliability = 0.6215, 2 blinded researchers).

**Table 5:** Factors Influencing Appropriateness of a Consult and Select Quotes

Theme	Major subthemes	Frequency	Quotations
Educational factors	Lack of insight into other specialties, especially outside of medicine	8/14	“No one truly knows what anyone else does. No one knows what it means to be in other specialties.” (INT 9) “As an internist, there are lots of areas we know something about, but we know exactly what we don't know… we see the big picture.” (INT 1) “I'm interested to learn, but we often get absorbed in our own things. There's no ownership or investment… they're not our patients.” (INT 8)
	Distance from common medical school training	6/14	“If everybody could remember what they learned from med school that would be dramatically helpful.” (INT 14) “In IM, you're continuing to practice what you learned in medical school, but for some specialties it might have been a while since touching those other areas.” (INT 1)
	Medical intern year	4/14	“Neuro does the same thing though—they often don't need our help—they did a full year of medicine, so they should know! They have a basic level of medicine.” (INT 5) “We took 1 year of medicine, so we know a little bit of their training, and the least they can do is do their part. It's frustrating that they don't do this most of the time. Other services are not trained in neuro exams. It's not right.” (INT 9)
Communication factors	Good consults must have a specific question	14/14	“Focus on what you need help with. If you're specific, you will get something specific in return.” (INT 1) “Consults should be a back-and-forth dialogue. There should be a clear, specific question and pertinent information. You should share your idea of what's going on and why you need help.” (INT 2)
	Consults should also be an educational opportunity	13/14	“Some consulting physicians provide recommendations, which serve as an opportunity to learn their thought process. It's important though to make the time and space, but that's difficult when we have so many patients.” (INT 1)
	Importance of follow-up	6/14	“The best consultants call after to follow-up and make sure thoughts and plans are conveyed in a conversation. But this requires initiative and is often not done, especially because doing the note in the chart is the bare minimum.” (INT 1) “More often, specialties are asking medicine for help with their question, but they won't follow-up. They just assume we'll take care of the patient and they can be done with everything.” (INT 7)
	Prior workup or perceived effort before calling a consult	6/7 Neurologists	“The team should have done some of the legwork on their own before the call. It's so frustrating when they haven't done anything and expect you to do it all.” (INT 6) “There is no reason why medicine can't do a history, a workup, a differential, and start things like understanding metabolics and possible infections. The majority of our consults are inappropriate as a result because they don't do their part.” (INT 9)
Clinical factors	Ability to manage a case	7/14	“Half the time, the person calling a consult is simply not confident in their abilities to perform, say, a neuro exam. And it may in fact be appropriate for them to not be so confident.” (INT 6) “It can be frustrating to find out how little people know. You don't know the true emergencies until you see the patient yourself.” (INT 9) “The expectation is that ER [emergency room] sees dizziness multiple times a day and calls for difficult cases. Sometimes I find that they just call for every patient because it's easier to have neuro do it for them or don't want to be held responsible.” (INT 13)
	Complexity of case	5/14	“So [it's] better to have both sides' input. In complex cases, it's not bad to consult. It is harder to keep track of and more work, but it should be done.” (INT 13) “Now I understand why I get some of the consults that I get—this ambiguity can be troubling if you don't know the area.” (INT 9)
Administrative factors	Attendings consider liability and covering yourself legally in consult decision-making	13/14	“When attendings tell you to call, you do it… probably to cover your [self]… and for legal/liability reasons. They want to be sure and don't want to miss things, even if they know what's going on.” (INT 5) “Liability is not what we're thinking about, but it's what senior people are thinking about. They want to cover their bases…” (INT 1)
	Cultural and incentive differences between institutions or departments	8/14	“We're also guilty in medicine—we need to communicate better. These different standards among specialties also create barriers and present missed educational opportunities…” (INT 3) “[A]ll the educational pushes haven't addressed the fact that different departments have different cultures, so we need to be sensitive to that when working together.” (INT 4) “All-department [interdepartmental] M&M meetings should occur when something bad happens… We need to talk with each other to make things move forward and so we can learn.” (INT 7) “Turf wars are a major part of this—people do not see consults due to perceived boundaries of their specialties.” (INT 5)
	Workflow challenges	6/14	“[W]e get consulted for things that are not an inpatient problem. Leaving this unaddressed will not affect patient care and is not important now. It can be dealt with after discharge, but we delay discharge, order extra tests, and call consults.” (INT 2) “I don't like the transfer to medicine service consults. It's not a medical consult but a management issue—it's about dumping patients.” (INT 5)
	Stress and workload	6/14	“Everyone is equally overworked and underpaid.” (INT 9) “It's harder to provide optimal patient care and a great learning environment when there is a lack of reimbursement and payment for consults and such high workloads.” (INT 2) “Some services are simply busier, so they will be more resistant.” (INT 1) “Attendings would never reject consults, but residents and fellows would. Probably because we are not incentivized for workload. It all stems from aggravation from being overworked.” (INT 5)

Overall, interviewee sentiments corroborated the notion of the dual fallacies:

No one truly knows what anyone else does. (INT 9)

I am usually open to consults. [But] sometimes I have to tell people, “This is your job!” (INT 5)

If everybody could remember what they learned from med school, that would be dramatically helpful. (INT 14)

It can be frustrating to see how little people know. (INT 6)

As an internist, there are lots of areas we know something about, and we know exactly what we don't know because we see the big picture. (INT 1)

### Semistructured Interviews: Common Qualities in Good Consults

Residents consistently reported common qualities in good consults. They unanimously independently volunteered that an appropriate consult must pose a specific question:

A good consult has to have a clinical question that is answerable. The referring physician should talk about the pertinent issues facing this patient and provide some detail. (INT 6)

Furthermore, almost all neurologists expected internists to complete an initial investigation before requesting a consult. However, residents unanimously reported that this never happens:

[T]he team should have done some of the legwork on their own before the call. It's so frustrating when they haven't done anything and expect you to do it all. (INT 8)

Residents understood that education would be an ideal secondary outcome from a consult, but high patient volumes and workload hinder it:

Well, there should be [an expectation of teaching and learning] but this is not always the case. I try to make conscious efforts to write out my thoughts and thought process so others can follow. When I am a consultant, I write notes in a way to teach so I don't get repeat[ed] consults for the same problem, but I can expect to get the same consults the next week. People tend to not be so receptive. (INT 7)

### Semistructured Interviews: Common Qualities in Inappropriate Consults

Residents also consistently reported common qualities of inappropriate consults. They perceived liability fear and overwhelming workloads as drivers for requesting inappropriate consults. Although the interviewees did not feel this pressure themselves, they unanimously identified attendings' fear of liability as a reason for calling consults:

Consults are called when we don't want to think about it and just want to cover our [selves]…. Residents don’t think about this, but attendings do. (INT 2)

Residents also identified that workload contributes to missed educational opportunities and a phenomenon of using consults to dump patients:

Bad consults… don't do workups and just try to dump patients on you. It's like they're outsourcing the management of their patient to you instead of performing efficient patient care. (INT 6)

### Semistructured Interviews: Proposed Solutions

Nearly all interviewees agreed that the aforementioned factors influenced consult quality, but interviewees' perspectives varied on possible solutions (Table 6). These were coded as technological, educational, and administrative solutions to consultation factors which participants previously identified. Technological solutions included leveraging the power of using an electronic medical record (EMR) for data transparency, shared decision-making, and communication:

We need systematic protocols for consults… maybe putting it in writing, maybe even all in the EMR. These would prompt specific questions and enforce closure so you hear back on the plan. (INT 5)

**Table 6:** Potential Solutions to Address Inappropriate Consults and Select Quotes

Theme	Major subthemes	Frequency	Select quotes
Technological solutions	Leveraging the electronic medical record (EMR)	12/14	“It's hard to gather… who is the consulting physician—what if there was a line in Epic [EMR] that showed the multiple teams involved and the contact information for the consultant?” (INT 8) “We need systematic protocols for consults… maybe putting it in writing, maybe even all in the EMR. These would prompt specific questions and enforce closure so you hear back on the plan.” (INT 5)
	Enhance how teams communicate through telecommunication and cell phone service improvement	5/14	“Some services don't respond and are hard to page. Sometimes, a person has the direct cell number of a surgeon, it makes a big difference.” (INT 8) “There has to be a better way to relay information to each other than paging them… [it] is archaic and inefficient.” (INT 7)
Educational solutions	Enhance rotations on other services to emphasize interdisciplinary collaboration	9/14	“The calls after consults to explain the notes and recs provide an important avenue for education—what if we had the medicine rotators who are on neuro for a few weeks actually learn what information is helpful for both parties involved?” (INT 9) “I know neurosurgeons spent a month with neuro—endless training of 7 years. But the attendings that did this saw the relationships between departments—less adversarial and more collegial… residents [should be] changing shoes for learning experiences. Colleagues do not have to be at odds with each other.” (INT 11)
Administrative solutions	Streamline workflows and protocols with regard to particular clinical scenarios	8/14	“We have 2 separate consults services for stroke and other consults—for other departments it seems so disjointed… Let's streamline consults within our own departments.” (INT 9) “It's also confusing how there are so many different subservices, so this should be streamlined.” (INT 6)
	Attending-directed services for noneducational consults	3/14	“Pulm[onology] does non-teaching consults. The attending triages all and does [consults] him/herself if [they are] not educational.” (INT 9) “Have a neurologist in the ER to triage and teach.” (INT 13)

Frequency: number of participants who reflected corresponding subthemes, of the 14 participants who were interviewed.

Educational solutions included efforts to have shared conference/didactic time between specialties, thoughtful rotations on other specialties with attention to common consultations, and creating a culture of learning:

I know neurosurgeons spent a month with neuro—endless training of 7 years. But the attendings that did this saw the relationships between departments—less adversarial and more collegial… residents [should be] changing shoes for learning experiences. Colleagues do not have to be at odds with each other. (INT 11)

Many felt that bridging communication and cultural issues between departments could reduce inappropriate consults:

All the educational pushes haven’t addressed the fact that different departments have different cultures, so we need to be sensitive to that when working together. (INT 4)

Finally, participants identified administrative solutions. These spanned collaborative development of clear workflows and protocols for particular patient presentations, streamlining the administrative burden of consultation, and development of attending-directed services for noneducational consults. These sought to alleviate administrative factors that detracted from the educational and patient-centered goals of interdisciplinary consultation:

Pulm[onology] does non-teaching consults. The attending triages all and does [consults] him/herself if [they are] not educational. (INT 9)

## Discussion

The results of this study suggest discordant expectations of expertise between neurology and medicine residents, which persisted despite implementing an educational intervention (neurology rotation for internists). These discrepancies support our hypothesis that both neurologists and internists believe that their own specialized knowledge is common, whereas unfamiliar information is beyond their scope—a cognitive bias that we have coined dual fallacies. These dual fallacies may impede interdisciplinary communication and collaboration in clinical practice.

In both phases, both specialties answered more questions correctly from their own specialty than the other group. This served as validation that the questions were appropriate for each specialty and matched their expertise. A core quantitative finding was that internists and neurologists both answered more medicine than neurology questions correctly, likely given the foundational nature of medicine questions and the shared training in medicine. Neurologists unsurprisingly answered more neurology questions correctly than internists. More internists than neurologists felt that their programs provided more education in other specialties, likely because internists spend more time learning foundational medicine across a broader spectrum of disciplines. Both neurologists and internists endorsed that they should know more neurology content then they actually do. This suggests a shared expectation of continual learning of specialized content.

However, other quantitative findings did not align with our hypothesis. Differences in perceived consult appropriateness were only statistically significant in phase 1 for internal medicine consultation (Table 2). Still, the tendency for internists and neurologists to identify respective internal medicine and neurology questions as their knowledge and thereby appropriate for consultation validates our other findings. Internists were more likely than neurologists to state that internists should know neurology answers and that neurologists were less likely to agree than internists on whether a case merited a medicine consult. Perhaps neurologists have reduced expectations of what internists should know, whereas internists may feel greater responsibility to cover more domains. Neurologists may also expect themselves to manage general medicine problems in addition to neurology-specific ones; this is reflected by neurologists rating they should know medicine answers more than internists felt that neurologists should. Alternatively, the shared year of internal medicine training could give neurologists a sense of false confidence in knowing more internal medicine than they actually do, whereas internists' additional years of internal medicine training expose them to what neurologists may not have learned. This is supported by the finding that internists were more accurate at assessing self-knowledge. Consistent with the latter part of the dual fallacies, this has implications for consultation—although inappropriate consultation may be wasteful, more harmful may be when a resident physician feels overly confident and does not consult when appropriate.

Semistructured interviews elucidated qualities of ideal consults. They also helped identify a diverse swath of potential individual- and system-level interventions to improve consultation (Table 6). Interviewees universally believed that consults must pose a specific question. This is concordant with our dual fallacies hypothesis; residents feel frustrated because they perceive the requester knows little, passes responsibility unnecessarily, is miscalculating the urgency of the consult, or is applying insufficient effort. More neurologists than internists believe that the primary team should complete preliminary investigations before calling consults. Neurologists may expect that internists know more neurology than they actually do and thus should be able to conduct preliminary investigations or feel that the preliminary investigation is insufficient because of unclear boundaries in scope of knowledge and practice.

What are the implications of these dual fallacies among physicians, particularly neurology physicians in training? Although the neurology rotation educational intervention did not fully close the gap in discordant expectations, our data from the surveys and interviews present several other intervention opportunities. These include incentivizing intellectual interest in other disciplines, clearly articulating scope of practice, and addressing frustration by chipping away at the dual fallacies. In practice, these may include postconsultation debriefs, improved consult cards informed by dual fallacies (to align expectations of knowledge base), and preliminary workup checklists for common consultation scenarios. As previously investigated by our research team, medicolegal liability may play a major role that deserves further investigation.^10^ Of interest, proposed solutions invoked larger-scale educational, technological, and administrative interventions, not merely reflecting on the clinical rigor of cases. This underscores how structural components of medical training can impede optimal consultation and trainee education.

Our data suggest that a neurology rotation for internists affected their self-perceived knowledge, actual knowledge on board-style questions, and perceptions of specialty knowledge. In phase 2 of our study, all internists had already rotated on the neurology consult service, thus granting them exposure to neurology's specialized care approaches and opportunities to work directly with neurologists. This may explain why neurologists also had a greater understanding of the extent of internists' knowledge. The phase 2 internist cohort had curricular exposure to neurology not experienced by the phase 1 internist cohort, evident in approximately 20% more internists self-rating their neurology knowledge as good. Most promising is that after the educational intervention, internists were more knowledgeable, felt more knowledgeable, and had higher expectation for themselves of knowing specialized neurology content than before the intervention.

This study was limited by its small sample size, response rate, and convenience sampling from 2 medical specialties at 1 institution. Our utilization of constructed common consultation scenarios written as board-style questions may not reflect the full scope of clinical situations faced by consulting providers. However, our novel methodology highlights ways of assessing biases and specialty knowledge through both self-rated and more objective means. Phase 1 and phase 2 cohorts were different samples; however, residency year and gender identity were not associated with any results, permitting us to draw conclusions through comparison. Although perceptions of inappropriate consults may change as physicians progress in their careers, we found that the inclusion of PGY-2s during phase 2 still resulted in discordant expectations. Although findings were largely consistent between phases, continued investigation into survey validity will encourage this methodology in future research and for other specialties.

Other limitations include potential biases of the researchers and methods used in collecting the qualitative data. Specifically, our qualitative data may be subject to selection bias due to participants' self-selecting whether to participate in the semistructured interview, as well as social desirability bias where participants may falsely wish to appear more collaborative, focused on education and patient care, and immune to negative perceptions of other specialties. In qualitative data analysis, 1 researcher served as the interviewer to standardize the interview experience, and transcripts were created verbatim from audio recordings immediately following interviews to virtually eliminate recall bias. However, it is possible that the qualitative coders, who were members of the research team, applied their own expectations to interpretation of data such that they classified data as consistent with overarching themes of interest. We sought to limit such biases by blinding 2 coders in categorizing interview subthemes and blinding a third coder in categorizing themes and reconciling discrepancies. In addition, although internal medicine senior residents and our institution's internal medicine program director were involved in the development of the instrument measures, board-style questions, and recruitment, the study team authoring this paper does not include an internist, which represents a potential bias in qualitative data interpretation. The first author's identity as an emergency medicine physician reflects the perspective of a generalist in medicine who similarly places neurology consults. Future studies on this topic should include interdisciplinary qualitative data reviewers.

Further work could explore other factors that influence specialty-dependent perceptions and interventions to reduce these differences. Some factors include time elapsed from core training and consults between specialties with less overlap than internal medicine and neurology (e.g., orthopedics and ophthalmology). Finally, the drivers of discordant perceptions evaluated in this study motivate interventions to reduce the perception of inappropriate consults and increase effective teaming. Residents in both fields agreed that communication is key; many see cross-pollination of expertise via rotations on other services as beneficial experiences to mitigate knowledge and communication issues, suggesting an appetite for interventions during residency to fill knowledge gaps and reduce misaligned perceptions. Furthermore, this study focused on 1 early-career, residency intervention whose effect may weaken across the lifespan of a medical career. This study also focused exclusively on residents; future directions for this work could include surveying neurology and medicine faculty to assess their perceptions of consultation appropriateness. They may help improve the validity of our results, especially given that a driver of consults perceived as inappropriate may stem from attendings' medicolegal concerns. Future research can investigate opportunities for continuing interdisciplinary medical education that sustainably reduce discordant consult perceptions. At our institution, conversations regarding consult appropriateness and cognitive biases have informed continued development of rotations on other specialties with explicit conversation and debrief with respect to interdisciplinary collaboration, shared didactics/conference educational sessions to discuss a coordinated approach for various patient presentations, and even transformations such as giving residents dedicated work phones—resulting in the sharing of direct lines to consultants and yielding a culture of accessibility and communication.

This pilot study suggests that biases in interdisciplinary consultation stem from discordant perceptions of knowledge familiarity and scope, consistent with cognitive and group psychology research. Our results indicate that residents of both neurology and internal medicine expected all residents to have specialized neurology knowledge, and both neurologists and internists agreed that internists should know more neurology than they do. Nevertheless, differing perceptions between the groups on consult appropriateness reflect these underlying beliefs. Nonclinical factors, ranging from cognitive biases to medicolegal and contextual considerations, substantially affect clinical decisions and patient care. Interventions, such as rotations with other clinical services, shared didactics, analytics to elucidate common consultation scenarios, and clear clinical consultation protocols, have become implemented at our institution and others. Although our neurology rotation intervention was insufficient for closing the gap in discordant knowledge expectations, residents in both disciplines identified additional interventions to foster cultures of learning. These interventions can help mitigate deeply rooted cognitive biases to empower neurology and non-neurology resident physicians to care for patients collaboratively in interdisciplinary teams.

## Appendix Authors

**Table:**

Name	Location	Contribution
Charles Sanky, MD, MPH	Department of Emergency Medicine, and Department of Medical Education, Icahn School of Medicine at Mount Sinai, New York, NY	Drafting/revision of the manuscript for content, including medical writing for content; major role in the acquisition of data; study concept or design; and analysis or interpretation of data
Caroline Gentile, MD	Department of Neurology, University of Pennsylvania, Philadelphia	Drafting/revision of the manuscript for content, including medical writing for content; major role in the acquisition of data; and analysis or interpretation of data
Jennifer Ren, BA	Department of Medical Education, Icahn School of Medicine at Mount Sinai, New York, NY	Drafting/revision of the manuscript for content, including medical writing for content, and analysis or interpretation of data
Eric Bortnick, MD	Department of Medical Education, Icahn School of Medicine at Mount Sinai, New York, NY	Drafting/revision of the manuscript for content, including medical writing for content; major role in the acquisition of data; and study concept or design
Stephen Krieger, MD	Department of Neurology, Icahn School of Medicine at Mount Sinai, New York, NY	Drafting/revision of the manuscript for content, including medical writing for content; major role in the acquisition of data; study concept or design; and analysis or interpretation of data

## Glossary

EMR: electronic medical record
PGY: postgraduate year
